# Corticosteroids vs. corticosteroids plus cycloporin A in adult minimal changes disease

**DOI:** 10.1186/1756-0500-2-144

**Published:** 2009-07-22

**Authors:** Dimitrios S Goumenos, Pantelitsa Kalliakmani, Eirini Savvidaki, John G Vlachojannis

**Affiliations:** 1 Internal Medicine-Nephrology, University Hospital of Patras, 26500 Patras, Greece

## Abstract

**Background:**

Adult minimal changes disease (MCD) is usually treated by high corticosteroids dose in order to achieve remission of nephrotic syndrome. In this study, the administration of high steroid dose (prednisolone 1 mg/kg BW/day) is compared with the combination of lower prednisolone dose (0.3 mg/kg BW/day) and cyclosporine A (CsA) (2–3 mg/kg BW/day) in a small number of patients.

**Findings:**

Eighteen patients were allocated to either prednisolone monotherapy or prednisolone and CsA combination, according to the risk of developing steroid side-effects. Complete remission of the nephrotic syndrome was observed in all patients treated by steroids or combination of steroids and CsA. Complete remission occurred in 67%, 89% and 100% of patients after 4, 8 and 12 weeks of treatment. Relapses occurred in 50% of patients from both groups, treated with the combination of low prednisolone dose and CsA and followed by sustained remission. Corticosteroidal side effects were observed only in high prednisolone dose (accumulated dose: 92.7 ± 22 mg/kg/BW vs. 58.5 ± 21 mg/kg/BW, p = 0.004).

**Conclusion:**

Treatment of adult MCD with low prednisolone dose and CsA seems to be equally effective with high prednisolone dose to induce remission of nephrotic syndrome. It is also effective as maintenance therapy for prevention of relapses and less frequently followed by corticosteroidal side effects.

## Background

Minimal changes disease (MCD) represens the most common cause of nephrotic syndrome in children accounting for more than 70% of cases, but it 's also the cause of adult nephrotic syndrome in 10 to 25% of cases [1,2]. The pathogenesis remains unknown. A shift of the T-cell repertoire, towards a Th-2 phenotype leading to release of putative permeability factors, is postulated [3]. Corticosteroids restore the glomerular expression of dystroglycans, adhesion molecules of foot processes, that are reduced in MCD patients with nephrotic syndrome [4,5].

Complete remission of proteinuria occurs in 90% of corticosteroid-treated children, early after initiation of treatment [6]. However, this period is longer in adults, whereas a large percentage of them shows relapses or becomes steroid-dependent [7]. Longer duration of treatment with high steroid doses increases the risk of serious side effects [2,8,9]. Cyclophosphamide and chlorambucil have been used in patients with frequent relapses, as maintenance therapy to prevent relapses and resulted in sustained remission [7], while the use of azathioprine and mycophenolate mofetil is rather limited [10].

CsA is used in patients with MCD, who continue to relapse after a course of alkylating agents, in cases with primary steroid resistance and recently as an initial treatment in children with steroid-sensitive nephrotic syndrome, in order to reduce the number of relapses [11]. However, CsA has not been used as an initial treatment in adult MCD. In this study the effect of prednisolone monotherapy (1 mg/kg BW/day) is compared to that of combination of lower prednisolone dose (0.3 mg/kg BW/day) with CsA, given in a small number of patients with MCD and a risk to high steroid dosage (obesity, borderline diabetes mellitus, osteoporosis and history of peptic ulcer).

## Methods

### Patients

Eighteen adult patients (8 males and 10 females) with nephrotic syndrome (urinary protein: 12 ± 6 g/24 h), due to biopsy-proven minimal changes disease, and well preserved renal function (baseline serum creatinine: 0.97 ± 0.3 mg/dl), referred to our department between 1998 and 2005, were included in the study. The inclusion criteria were age between 18 and 65 years old, urinary protein >3.5 g/24 h and creatinine clearance >60 ml/min. The clinical and biochemical features of patients at presentation are shown in table 1.

**Table 1:** Clinical and histological features of patients treated by steroids or combination of steroids with CsA, at presentation.

**Feature**	**Steroid** **(n = 10)**	**Steroid+CsA** **(n = 8)**	**P**
Gender (M/F)	5/5	3/5	NS
Age (years)	39 ± 14	44 ± 18	NS
Blood pressure (mmHg)	135/83	137/86	NS
Serum creatinin (mg/dl)	0.95 ± 0.4	1.0 ± 0.2	NS
Albumin (g/L)	23 ± 3	22 ± 6	NS
Urinary protein (g/24 h)	10 ± 5	9 ± 9	NS
Glomerulosclerosis (%)	10 ± 7	10.5 ± 4	NS
Interstitial fibrosis (absent/present)	8/2	7/1	NS
Interstitial edema (absent/present)	0/10	0/8	NS
Tubular atrophy (absent/present)	8/2	5/3	NS
Vascular hyalinosis (absent/present)	6/4	5/3	NS

All patients were treated by either prednisolone alone or combination of lower prednisolone dose with CsA (Neoral). The latter was given to patients with obesity (body mass index >30 kg/m^2^), fasting blood glucose between 100 and 120 mg/dl, osteoporosis and peptic ulcer, diagnosed by endoscopy, in order to avoid high prednisolone doses. Ten patients were treated with prednisolone monotherapy (initial dose 1 mg/kgBW/day) and eight patients with lower prednisolone (0.3 mg/kgBW/day) and cyclosporine (2–3 mg/kg BW/day). The dose of CsA was adjusted to maintain trough blood levels of 100 ng/ml for 12 months, mean CsA dose: 2.07 ± 0.5 mg/kgBW/day, gradually reduced to 0.5 mg/kg BW/day per month. Prednisolone was also gradually reduced and discontinued after 6 months, at least. The two different treatment protocols are presented in table 2. The mean duration of treatment with prednisolone was 13 ± 5 months and that of cyclosporin 23 ± 10 months. The total accumulated dose of corticosteroids were significantly higher in patients treated by high steroid dose compared to the combination treated group (92.7 ± 22 mg/kg vs. 58.5 ± 21 mg/kg, p = 0.004) (table 2).

**Table 2:** Treatment protocol in patients treated with steroid or combination of steroid with CsA

**Time period**	**Steroid** **(n = 10)**	**Steroid+CsA** **(n = 8)**
	**Prednisolone dose** (mg/kg/day)	**Prednisolone dose** (mg/kg/day)
1^st ^month	1	0.3
2^nd ^month	0.6	0.15
3^rd ^month	0.3	0.10
4^th ^month	0.15	0.07
5^th ^month	0.07	0.07
6^th ^month	0.07	0.07
	on alternate day	on alternate day
		
Mean accumulative prednisolone dosage (mg/kgBW)	92.7 ± 22	58.5 ± 21
		
Mean CsA dosage for target trough levels 100 ng/ml (mg/kgBW/day)	-	2.07 ± 0.5

Patients from both groups, who showed relapses of the nephrotic syndrome, were treated with low prednisolone (0.3 mg/kg BW/day) and CsA (2–3 mg/kg BW/day), regardless of initial treatment, in order to avoid the administration of higher prednisolone doses.

### Conventional histopathology and grading of histopathological involvement

The diagnosis was made by renal biopsy on light-microscopy (LM) and immunoflurescence (IF). Minimal changes was diagnosed by the presence of normal glomeruli or mild mesangial matrix increase and focal mesangial hypercellularity in the LM [8]. Lack of immune deposits and complement components, as well as presence of scattered mesangial IgM deposits, were the main features in IF.

The degree of glomerular sclerosis, interstitial edema and fibrosis, as well as tubular atrophy and arteriolar hyalinosis, were evaluated from Masson's trichrome stained sections of each biopsy. The severity of glomerular sclerosis was expressed as the percentage of totally sclerosed glomeruli, whereas other parameters were evaluated by a semi-quantitative method and expressed as present or absent [12].

### Follow-up – Definition

During treatment and follow-up period, all patients were examined every month. Body weight, blood preassure, urinalysis, full-blood count, biochemical profile and 24 h urinary protein were recorded. Blood pressure control, (135/85 mmHg) was achieved by ACEi, calcium-channel blockers, beta-blockers and diuretics in both groups of patients.

Remission of nephrotic syndrome was defined as complete or partial if proteinuria was <0.3 g/24 h and between 0.3 and 3 g/24 h respectively. Relapse was considered when edema and proteinuria >3.5 g/24 h re-appeared, in patients with remission. The follow-up period was 6 ± 3 years. The development of chronic renal failure was estimated using the end-points of 50% and 100% increase (doubling) of base-line serum creatinine.

### Statistical analysis

Variables are presented as mean ± SD. Unpaired t-test was used to compare baseline clinical and biochemical data, between the two groups of patients. Chi-square test for independence, was used to compare histopathological data and remission rate of proteinuria, in the two groups of patients. A p-value of less than or equal to 0.05 was considered to be statistically significant.

## Results

### Remission of nephrotic syndrome

**Figure 1:**
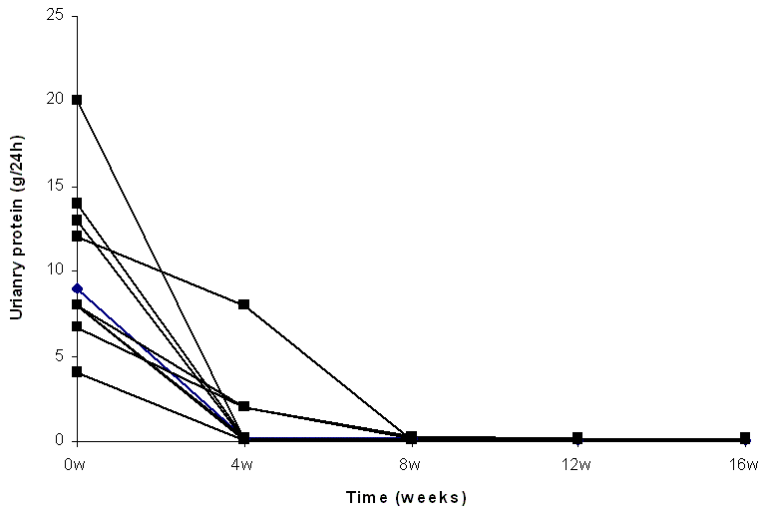
**Individual courses of the urinary protein loss in patients treated by high steroid dose**.

**Figure 2:**
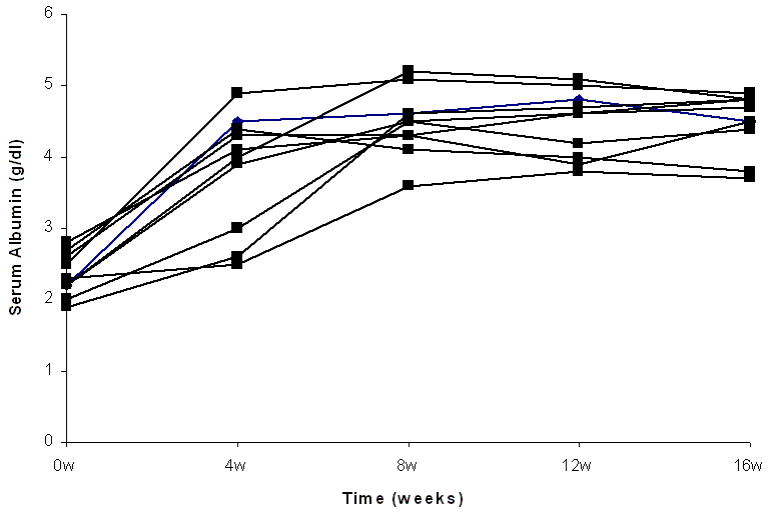
**Individual courses of serum albumin in patients treated by high steroid dose**.

**Figure 3:**
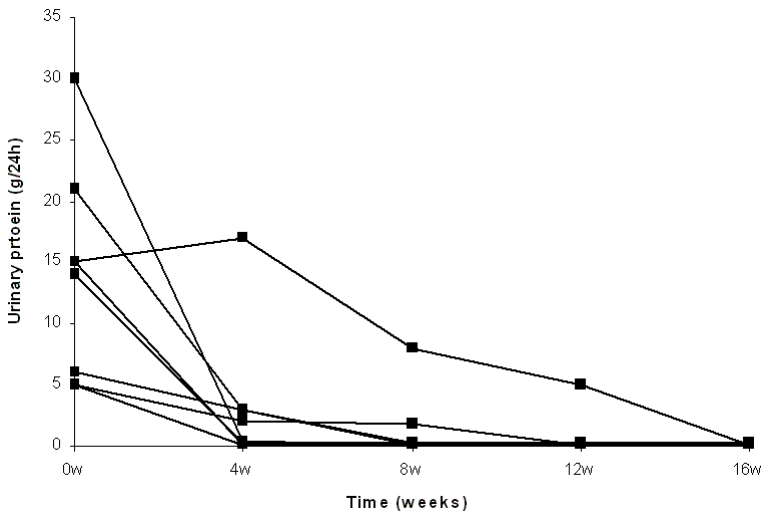
**Individual courses of the urinary protein loss in patients treated by lower steroid dose and CsA**.

**Figure 4:**
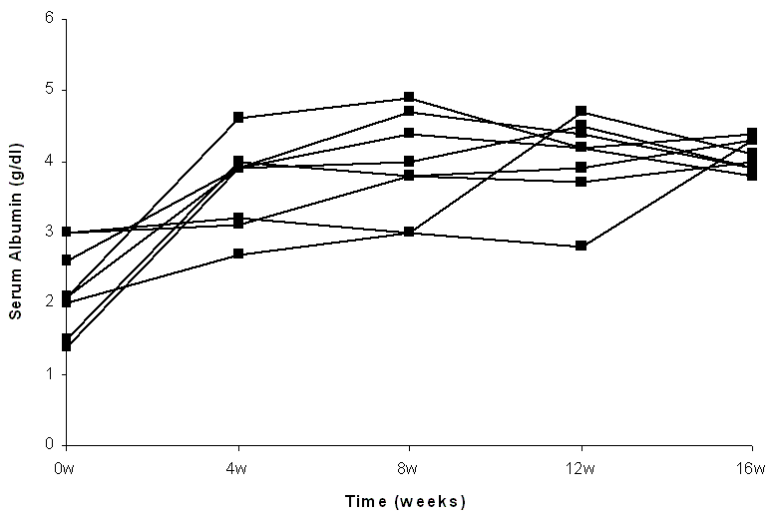
**Individual courses of serum albumin in patients treated by lower steroid dose and CsA**.

Complete remission of nephrotic syndrome was observed in all patients treated by either high steroid dose or combination of low steroid dose and CsA. The number of patients from both groups with complete remission after 4, 8 and 12 weeks of treatment was 12 (67%), 16 (89%) and 18 (100%) respectively. At 4 weeks, 7 patients from the steroid treated group and 5 from the combination treated group, showed complete remission (p = NS). At 8 weeks all steroid treated and 6 from the steroid and CsA treated group presented complete remission (p = NS), whereas at 12 weeks all patients from both groups were in complete remission (Fig. 1, 2, 3 and 4).

### Relapses of the nephrotic syndrome

Relapses occurred in 9 patients (50%), 4 initially treated with steroids and 5 with combination of lower steroid dose and CsA (p = NS). Seven patients showed the first episode of relapse, during tapering of steroid or CsA dose, whereas 2 showed relapse one and six months after discontinuation of treatment. All relapsers were treated by low prednisolone dose and CsA and showed complete remission. Two of 9 patients (22%) showed more than 2 episodes of relapse and treated for a longer period of time (40 and 36 months respectively), using a long-term low maintenance dose of CsA (1 mg mg/kg BW/day). Since renal function is not a reliable indicator of CsA nephrotoxicity and histological examination is necessary [13], these two patients underwent a repeat renal biopsy, in order to identify potential CsA nephrotoxicity [14].

### Clinical outcome after discontinuation of treatment

All patients preserved their renal function and showed no increase of serum baseline creatinine, by 50 or 100% during the follow-up period. All patients remained in complete remission of nephrotic syndrome three years after discontinuation of treatment that was given either for the first episode of nephrotic syndrome or for treatment of relapses.

### Side effects

Corticosteroidal side effects were observed only in patients treated with high prednisolone dose. Cushingoid was observed in all of them, striae in 4, diabetes mellitus in 2 and gastrointestinal intolerance in one. Patients treated with lower prednisolone dose showed no side effects related to corticosteroids.

Apart from hypertrichosis in 2 patients no other side-effects related to CsA were observed. In 2 patients with long-term administration of CsA, no typical lesions of CsA nephrotoxicity were observed in the repeat biopsies.

## Discussion

Steroids represent the cornerstone of treatment in MCD, but although most patients show complete remission, 31%–76% of them show relapses in various studies. High daily steroid dose followed by alternate-day administration, after remission of proteinuria, is used as an initial treatment in adult MCD. With this therapeutic approach, remission of the nephrotic syndrome occurs in 46%–62% at 4 weeks, 67%–87% at 8 weeks and 75%–100% at 16 weeks of treatment [2,8,9,15,16]. A similar response rate, was observed in this study with high prednisolone dose.

CsA has been used in patients with frequently relapsing, steroid-dependent and steroid-resistant idiopathic nephrotic syndrome [17,18]. Recently, CsA given in combination with prednisone, to children with steroid-sensitive nephrotic syndrome, was proved effective in reducing the number of relapses for the first year of follow-up [13]. The major problem, with the administration of CsA is its potential nephrotoxicity [14] and the frequent relapses after its discontinuation. For this reason, CsA was used at low doses and stopped after gradual tapering [19]. Combination of low prednisolone dose with CsA has been used as initial treatment in patients with focal segmental glomerulosclerosis and obesity, borderline diabetes mellitus or osteoporosis, in order to avoid higher steroid doses [20,21]. In this study, a similar regimen used in adult MCD patients with relative contraindications to the high steroid dosage was followed by complete remission in 63% and 100% of patients after 4 and 12 weeks respectively. Although the number of patients was small the results were comparable to those observed with high prednisolone dose.

A significant problem in patients with minimal changes disease is the relapse of the nephrotic syndrome within the first 12 months, after steroid withdrawal (31%–76%). In our study, relapses occurred in 50% of patients. Although no significant difference was observed in the relapse rate between the two groups, a trend towards more frequent relapses was observed with CsA (40% vs. 63%). Since prolonged high corticosteroid dose and administration of cytotoxic drugs are usually followed by serious side effects, we treated our patients with relapses, with a combination of low prednisolone and CsA. All patients showed stable remission, probably related to the long period of maintenance therapy.

The prolonged use of high corticosteroids dose is usually followed by significant side effects [2,8,9], a finding also confirmed in our study. However, patients treated with lower steroid and CsA dose, although treated for longer period of time, showed no side effects.

In conclusion, treatment of adult MCD with low prednisolone dose and CsA seems to be equally effective with high prednisolone dose, in the remission rate of nephrotic syndrome and is less frequently followed by side effects. However, further research including higher number of patients treated with low prednisolone dose and CsA, is required, in order to establish this regimen as initial treatment of adult MCD.

## Competing interests

The authors declare that they have no competing interests.

## Authors' contributions

This work was designed and supervised by DSG and JGV. Patient follow-up was recorded by PK and ES, while the statistical analysis was performed by PK
